# A caecal pseoudotumour with an incidental adenomatoid testicular tumour in a man with right undescended testis: a case report

**DOI:** 10.1186/s12957-016-0995-1

**Published:** 2016-09-01

**Authors:** Alex Muturi, Vihar Kotecha, Cynthia Ojee, Desmond Mang’oka, John Muthuri

**Affiliations:** 1 University of Nairobi, Nairobi, Kenya; 2 Kenyatta National Hospital, Nairobi, Kenya

**Keywords:** Pseudotumour, Adenomatoid testicular tumour, Cryptorchidism

## Abstract

**Background:**

Inflammatory pseudotumour refers to a non-malignant tumour-like mass resulting from an inflammatory reaction that is composed of granulation tissue with leukocyte infiltration that commonly occurs in the paediatric or young adult population. These tumours occur more commonly in the lungs and the orbit but rarely does it affect the gastrointestinal tract. It poses a clinical diagnostic challenge since it is a benign condition than can mimic the malignant counterpart. Our case is a rare presentation of the caecal pseudotumour in the presence of a right undescended abdominal testis evaluated as a caecal tumour with a differential diagnosis of a testicular malignancy.

**Case presentation:**

We report a 53-year-old male who presented with clinical signs suggestive of right colon tumour and undescended right testis. Intra-operatively, a caecal mass was found with no clearly discernable appendix and extensive adhesion of the right colon to the retroperitoneum, to the liver and gall bladder. A testis was found adherent to the posterior aspect of the caecum and terminal ileum. A right hemicolectomy was performed. Histopathology findings revealed an inflammatory mass with abundant fibroblast proliferation and chronic inflammatory cells infiltrate, involving bowel wall and periceacal adipose tissue; no malignant cells were identified. The testis had within it an adenomatoid tumour nodule. He had uneventful recovery and was discharged home 7 days post-operatively. At the moment, he is symptoms free.

**Conclusions:**

The occurrence of right colonic inflammatory pseudotumour and co-existent adenomatoid testicular tumour arising from a cryptorchid testis is very unusual. This would make one incline towards a malignant testicular lesion in the presence of cryptorchidism. Testicular adenomatoid tumour is a rare benign neoplasm, mostly affecting fully descended testis and usually does not warrant orchidectomy for purposes of preserving testicular function. On the other hand, surgical resection remains the only safe and curative treatment option available for inflammatory pseudotumours.

## Background

Inflammatory pseudotumour (IPT) is a rare benign tumour whose aetiology and pathogenesis are uncertain [1]. These tumours occur more commonly in the lungs and the orbit, additionally other sites affected include the major salivary glands, kidney, liver, omentum, ovary, larynx, urinary bladder, breast, pancreas, spleen, lymph nodes, skin, soft tissues, and central nervous system [2]. Although rare, IPT is important because its clinical and radiological findings mimic those of a malignant neoplasm [3]. IPT of the caecum is an extremely rare process, and this diagnosis tends to be given from an erroneous impression of malignancy of the right side of the colon based on a clinical picture of unexplained fever, weight loss or anaemia [4, 5].

Adenomatoid tumours are rare benign tumours of mesothelial origin affecting both male and female genital tracts. These tumours are more common in the male adnexa accounting for about 30 % of all paratesticular tumours [6]. It is incidentally found in autopsy specimens and surgical specimens of patients with cryptorchidism. It can be confused with malignancy, and it is therefore important to make the correct diagnosis to avoid unnecessary orchidectomy [6, 7].

We report a rare case of caecal tumour in a patient with right undescended testis harbouring testicular adenomatoid tumour.

## Case presentation

We report a 53-year-old mason, from rural Kenya who presented with right-side undescended testis and right lower quadrant abdominal pain for 1 year. The pain was insidious on the onset and progressed slowly; it was aggravated by feeding and activity and relieved by analgesics and rest. He had associated anorexia, 7-kg weight loss over 4 months and night sweats. He had no nausea, no change in bowel habit and no blood in stool. He denied history of exposure to pulmonary tuberculosis and gave no family history of cancer.

Upon examination, he was slightly wasted, afebrile, not pale, not icteric and no pedal oedema. The vital signs were within normal.

He had a normal abdominal examination except for an empty right hemiscrotum and no palpable mass along the inguinal canal. The left testis was normal in size and shape and within the left hemiscrotum.

Total blood count revealed a white blood cell (WBC) count of 9.98 × 10^9^/L, relative neutrophilia of 78.8 %, haemoglobin (Hb) 14.2 g/dl and platelet count of 378 × 10^9^/L. He had normal renal functions, electrolytes and liver functions.

**Fig. 1:**
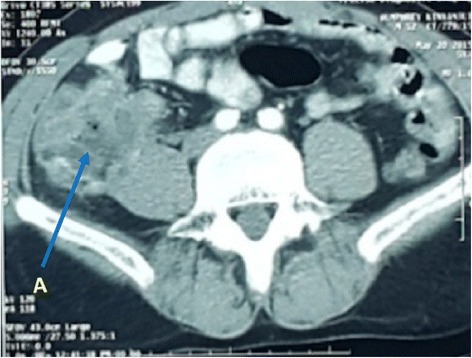
The *arrow A* shows irregular thickening of the caecal wall, maximum thickness reported as 30 mm, with no identifiable appendix, and the rest of the bowel were normal

Abdominal ultrasound revealed a right iliac fossa mass and could not visualise the appendix, giving an impression of appendicular mass. Contrast-enhanced abdominal CT scan (Fig. 1) showed irregular thickening of the caecal wall, maximum thickness reported as 30 mm, with no identifiable appendix, and the rest of the bowel were normal. It confirmed the presence of intra-abdominal right testis. Colonoscopy showed inflamed caecum with the rectum and the rest of the colon reported as normal. Histopathology of the specimen taken during colonoscopy showed chronic inflammation. The Mantoux tuberculin skin test was negative. Carcinoembryonic antigen, α-feotoprotein and β**-**HCG levels were normal.

**Fig. 2:**
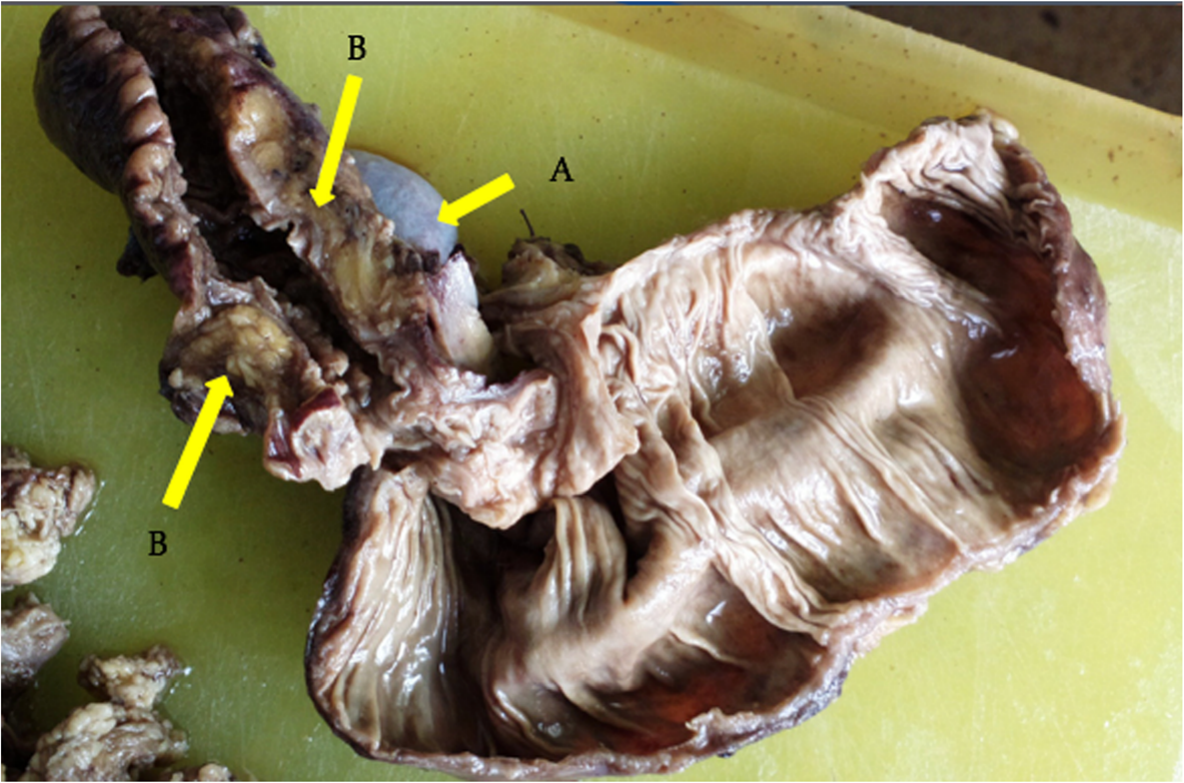
The cut surface of the entire specimen showed grossly normal tan white caecal mucosa, thickened terminal ileum (**b**) with an embedded testis (**a**)

Laparotomy was done, and we found a thickened caecum with extensive adhesions to the anterior abdominal wall with a discoid mass adherent to the retroperitoneal surface of the caecum. On close examination, the discoid structure was found to be the undescended testis with its epididymis. The appendix was normal in appearance. Right hemicolectomy was performed in view of the caecal thickening. Pathology report stated an ill-defined mass measure of 35 × 30 mm on the surface of the caecum that was tan brown and haemorrhagic (Fig. 2). The appendix was normal on gross inspection, and there was no gross mural invasion into the caecum. The cut surface of the entire specimen showed grossly normal tan white caecal mucosa with thickened terminal ileum (Fig. 2 arrow b) with an embedded testis (Fig. 2 arrow a).

**Fig. 3:**
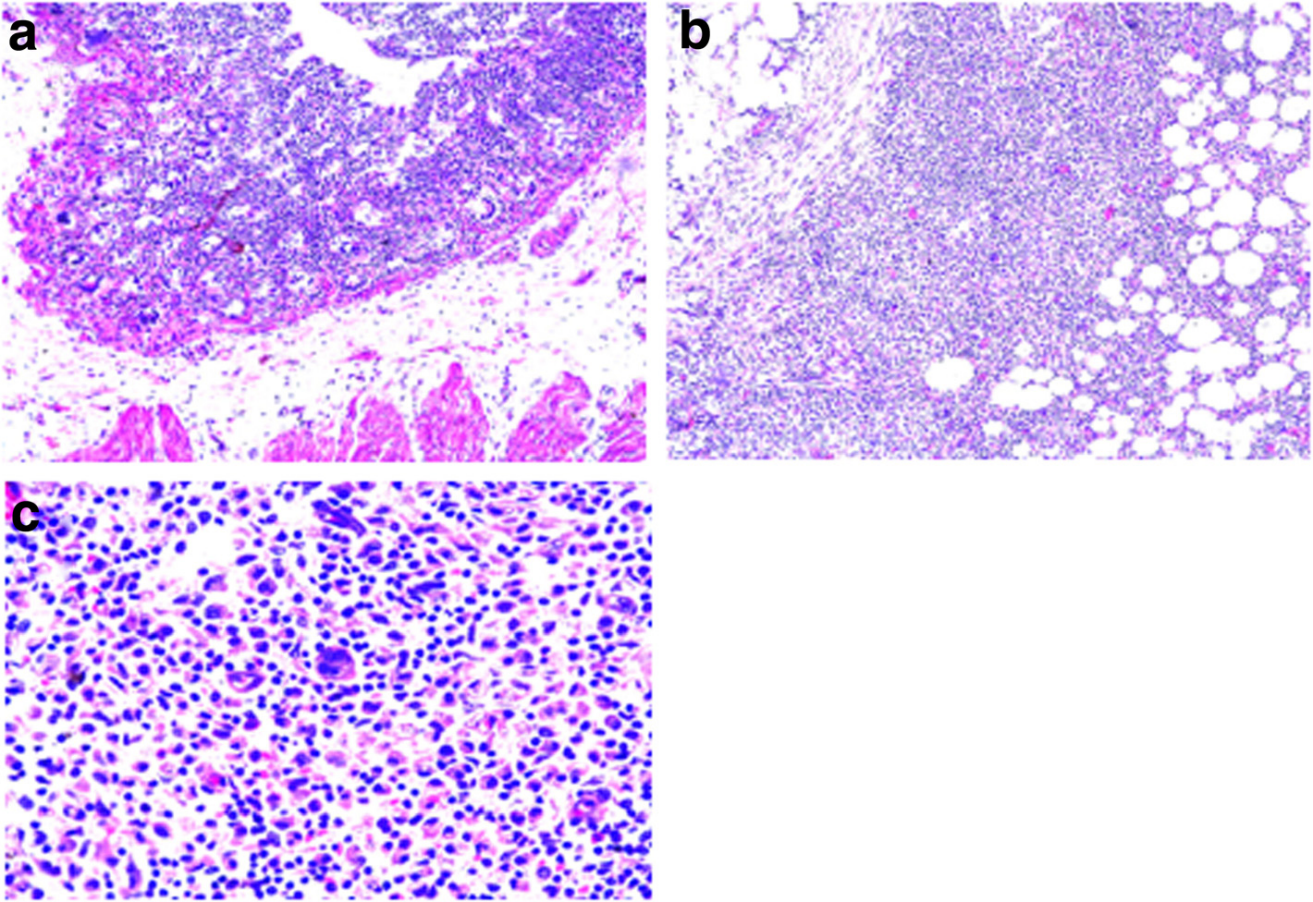
Caecal serosa and periceacal fat infiltrated by intense polymorphic chronic inflammatory cells with multinucleated giant forms. Panels **a** and **b** at lower power, panel **c** is of high power

**Fig. 4:**
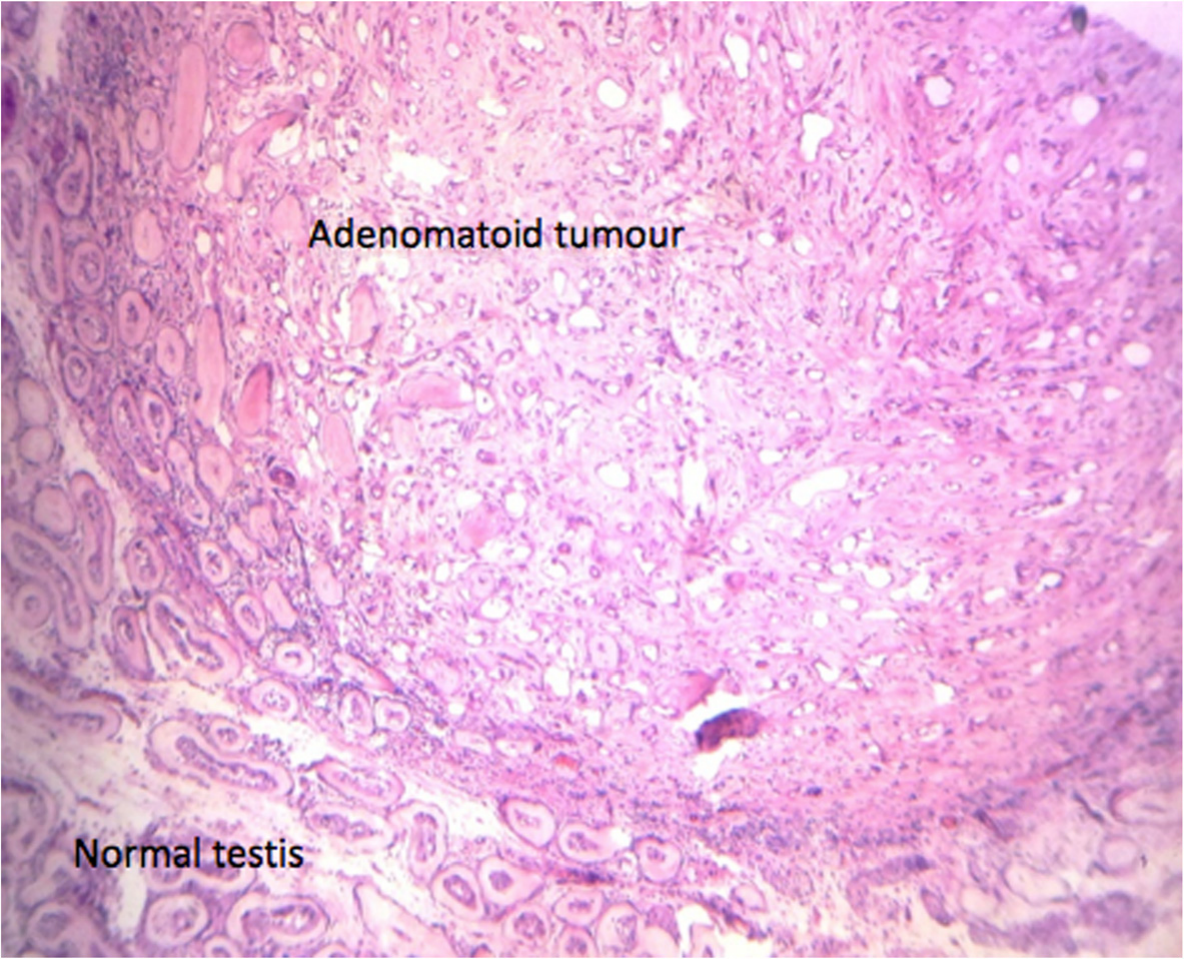
Nodular lesion of the testis encapsulated well-circumscribed lesion comprising of round to oval cells with multiple ectatic spaces (adenomatoid tumour)

Microscopically (Fig. 3a–c), the tumour showed diffuse serosa and periceacal fat infiltration by polymorphic population of chronic inflammatory cells comprising of lymphocytes, plasma cells, macrophages and occasional multinucleated cells. The mucosa, submucosa and muscularis propria were intact, with no features of malignancy or granuloma. There were pericolic lymph nodes that showed reactive changes. The vermiform appendix was normal. The testicular tissue was processed separately and showed global tubular hyalinization with thickened basement membrane and prominent leydig cells with an adjacent well-circumscribed uncapsulated nodular lesion exhibiting cuboidal to flat cells disposed in solid cords alternating with multiple channel simulating vascular structures (Fig. 4).

### Discussion

Inflammatory pseudotumour refers to a non-malignant tumour-like mass resulting from an inflammatory reaction and composed of granulation tissue with leukocyte infiltration, commonly occurring in the paediatric or young adult population [8, 9]. Our patient was a 53-year-old male and does not fit the expected age bracket for this condition.

The symptoms of IPT are nonspecific making the diagnosis challenging [10]. Our patient presented with intermittent abdominal pain, anorexia and weight loss raising suspicion of right colonic malignancy with a differential diagnosis of inflammatory conditions such as caecal tuberculosis.

There are nonspecific laboratory features for IPT such as elevation of white cell count, platelet count, C-reactive protein and immunoglobulin G (IgG) levels [11]. Some cases of IPT have been reported in the setting of IgG4-related sclerosing disease: a systemic disease manifesting as autoimmune pancreatitis, sclerosing cholangitis, cholecystitis, sialadenitis and retroperitoneal fibrosis [12]. The white cell count and platelet count of our patient were within normal limits. It is regrettable that the C-reactive protein and IgG levels were not done. The CEA, β-HCG and α-fetoprotein were all within normal; they were ordered to rule out testicular malignancy arising from the cryptorchid testis.

The radiological features of IPT are nonspecific, and this poses additional diagnostic challenge [13]. On ultrasound images, lesions can be hypoechoic or hyperechoic with either ill-defined or well-circumscribed edges. These lesions often have increased vascularity during colour on Doppler studies [14]. CT also shows variable appearances with lesions having low, equal or high intensity compared with the neighbouring tissues. On magnetic resonance imaging (MRI), IPT usually has low signal intensity on both T1- and T2-weighted images, which may be due to the fibrotic nature of these lesions [15]. For our patient, the abdominal ultrasound was reported as normal but for the fact that the appendix was not visualised. The contrast CT scan only showed hyperdense focal thickening of the caecal wall and the cryptorchid right testis. He did not have MRI.

Histologically, IPT contains a variable amount of acute and chronic inflammatory cells, i.e. lymphocytes and plasma cells and myofibroblastic spindle cells [16]. For this case, the microscopic examination of the caecal tumour showed fibrosis and chronic inflammatory cells but no evidence of malignancy or granulomas, therefore ruling out colonic neoplasm, and Ziehl-Neelsen (ZN) staining for caecal tuberculosis was also negative.

Although there have been reports that steroid therapy, nonsteroidal anti-inflammatory drugs and thalidomide shrinks pseudotumour, surgical resection of the mass remains the only curative option [16, 17]. Before surgery, IPT often cannot be differentiated clinically or radiologically from other more aggressive neoplasms, and the accurate diagnosis is based on histopathology examination. It is for this reason that colonic IPT is usually treated with the same oncologic and anatomic resection principles as colon cancer [11, 18]. Our patient underwent a right hemicolectomy because the tumour was involving the caecum. The inadvertent ureteric injury was due to the adhesions encountered whilst mobilising the right colon.

Adenomatoid tumours of the testis present as well-circumscribed, hyperechoic and solid masses usually between 1 and 5 cm on ultrasound examination [19]. Besides having undescended testis, our patient had no other complaints attributable to genital tract tumour. He had not sought medical attention for the undescended testis. The left testis was normal, and he is a father of three. The abdominal location of the cryptorchid testis was confirmed on contrast-enhanced CT scan. In view of his age and given the fact that it was an intra-abdominal testis, the best treatment option for him was orchiectomy, which was accomplished at the time of the resection of the caecal tumour. These tumours have a predilection for white males, occurring between the third and fifth decade with a mean age of 36 years and mainly affect the epididymis, spermatic cord, prostate and ejaculatory ducts [20]. Our patient had an unusual presentation given his age and the fact that it involved a cryptorchid testis. These tumours when they arise from cryptorchid testes are discovered incidentally at the histology of the submitted testicular tissue following orchiectomy or at autopsy [7, 21].

## Conclusions

The clinical features of IPT are nonspecific, and the diagnosis can be quite challenging. Surgical resection remains the only safe and curative treatment option available. Testicular adenomatoid tumour is a rare benign neoplasm, which when discovered, the need to preserve endogenous testicular function is key but not always practical especially for cryptorchid testes. Occurrence of right colonic IPT and co-existent adenomatoid testicular tumour arising from a cryptorchid testis is very unusual.

## Abbreviations

CEA: Carcinoembryonic antigen
β-HcG: Beta human chorionic gonadotropin
CT: Computed tomography
Hb: Haemoglobin
IgG: Immunoglobulin G
IPT: Inflammatory pseudotumour
MRI: Magnetic resonance imaging
WBC: White blood cell
ZN: Ziehl-Neelsen
